# Extracellular vesicles in Alzheimer's disease

**DOI:** 10.1055/s-0044-1779296

**Published:** 2024-03-11

**Authors:** Victor Hugo Berriel Pinho, João Paulo Lima Daher, Salim Kanaan, Thalia Medeiros

**Affiliations:** 1 Universidade Federal do Rio de Janeiro, Faculdade de Medicina, Rio de Janeiro RJ, Brazil.; 2 Universidade Federal Fluminense, Faculdade de Medicina, Departamento de Patologia, NIterói RJ, Brazil.

**Keywords:** Extracellular Vesicles, Central Nervous System, Alzheimer Disease, Biomarkers, Early Diagnosis, Signal Transduction, Vesículas Extracelulares, Sistema Nervoso Central, Doença de Alzheimer, Biomarcadores, Diagnóstico Precoce, Transdução de Sinais

## Abstract

Extracellular vesicles (EVs) are small vesicles released by cells that facilitate cell signaling. They are categorized based on their biogenesis and size. In the context of the central nervous system (CNS), EVs have been extensively studied for their role in both normal physiological functions and diseases like Alzheimer's disease (AD). AD is a neurodegenerative disorder characterized by cognitive decline and neuronal death. EVs have emerged as potential biomarkers for AD due to their involvement in disease progression. Specifically, EVs derived from neurons, astrocytes, and neuron precursor cells exhibit changes in quantity and composition in AD. Neuron-derived EVs have been found to contain key proteins associated with AD pathology, such as amyloid beta (Aß) and tau. Increased levels of Aß in neuron-derived EVs isolated from the plasma have been observed in individuals with AD and mild cognitive impairment, suggesting their potential as early biomarkers. However, the analysis of tau in neuron-derived EVs is still inconclusive. In addition to Aß and tau, neuron-derived EVs also carry other proteins linked to AD, including synaptic proteins. These findings indicate that EVs could serve as biomarkers for AD, particularly for early diagnosis and disease monitoring. However, further research is required to validate their use and explore potential therapeutic applications. To summarize, EVs are small vesicles involved in cell signaling within the CNS. They hold promise as biomarkers for AD, potentially enabling early diagnosis and monitoring of disease progression. Ongoing research aims to refine their use as biomarkers and uncover additional therapeutic applications.

## Resumo

As vesículas extracelulares (VEs) são pequenas estruturas liberadas pelas células que agem na sinalização celular. No sistema nervoso central (SNC), as VEs são estudadas em relação à doença de Alzheimer (DA), um distúrbio neurodegenerativo que cursa com declínio cognitivo e morte neuronal. As VEs podem ser biomarcadores potenciais para a DA devido ao seu papel na progressão da doença. As VEs derivadas de neurônios, astrócitos e células precursoras apresentam alterações na DA, contendo proteínas associadas à patologia da DA, como beta-amiloide (Aß) e tau. Níveis elevados de Aß foram observados nas VEs de neurônios de indivíduos com DA, sugerindo seu potencial como biomarcadores precoces. A análise de tau nas VEs de neurônios ainda é inconclusiva. Além disso, as VEs neurais carregam outras proteínas relacionadas à DA, incluindo proteínas sinápticas. As VEs podem ser promissoras como biomarcadores para o diagnóstico precoce e monitoramento da DA, porém mais pesquisas são necessárias para validar seu uso e explorar aplicações terapêuticas. Em resumo, as VEs são vesículas envolvidas na sinalização celular no SNC, com potencial como biomarcadores para a DA.

## INTRODUCTION

Extracellular vesicles (EVs) are defined as vesicles bounded by a phospholipid bilayer originating from endosomes or from the evagination of the plasma membrane that do not have the ability to replicate itself. 1 EVs can be classified according to different parameters, such as the mechanism of biogenesis or with respect to their size, even if the latter method of differentiation is not very effective, since an overlap between size ranges of different types of extracellular vesicles occurs. 1 2 3 4 Thus, recommendations from the International Society of Extracellular Vesicles suggest, when the origin is unclear, identifying ∼100-1000 nm vesicles as “large EVs” (lEVs) and ∼40-100 nm vesicles as “small EVs” (sEVs).

Regarding their biogenesis, EVs can be formed from the evagination of the plasma membrane, to form the so-called microvesicles (MVs) or microparticles (MPs), or they can be formed from the invagination of the membrane of endosomes, forming multivesicular bodies (MVBs) that contain in their interior exosomes, which are released from the fusion of the MVB with the plasma membrane. 2 3 Once in the extracellular *millieu* , EVs act as important cell signaling mediators since they carry several important molecules, such as proteins, lipids, carbohydrates, and genetic material. 5 6 In this sense, one can affirm that EVs may be involved with pathological processes and even serve as biomarkers of various diseases. In this review, we summarize the accumulating evidence that supports the idea of using EVs as biomarkers that could enable not only the diagnosis but also monitoring the progression of Alzheimer's disease.

## EXTRACELLULAR VESICLES IN CELLULAR COMMUNICATION, CENTRAL NERVOUS SYSTEM PHYSIOLOGY AND ITS ROLE ON THE PATHOPHYSIOLOGY OF DISEASES

EVs can be found in various biofluids, not only in blood, and their content may reflect both their origin and function. 7 Regarding function, it is thought that EVs may serve as a cell signaling mechanism, being involved in systemic processes, such as immune response and inflammation, as well as pathological processes, which has increased the interest in their potential as biomarkers of such diseases. 8 9 Colombo et al. 2014 10 demonstrated the participation of exosomes in processes such as angiogenesis, inflammation, transport of morphogens, and apoptosis. The EV release by central nervous system (CNS) cells, such as neurons, astrocytes, oligodendrocytes, and microglia, has been established for many years. 11 12 13 14 There are several studies that investigated some of the physiological actions that EVs play in the central nervous system, although most of them are in vitro or in animal models. It is established that active neurons secrete exosomes containing various contents, mostly proteins and genetic material, which act by altering the expression of proteins in the recipient neurons, as well as by modulating neurotransmission and even neurogenesis. 15 Moreover, other studies have already demonstrated that the internalization of EVs secreted by certain groups of neurons, by other groups of neurons, may affect the process of synaptic plasticity. 16 17 Neurons can also communicate with glial cells via EVs. For example, one study demonstrated that neuron-derived exosomes containing a family of miRNAs, when internalized by astrocytes, led to increased expression of several proteins, including glutamate-transporter-1(GLT-1). This protein plays a key role in maintaining the homeostasis of glutamatergic synapses. 12 18 When it comes to astrocytes, some studies point to important functions performed by the EVs secreted by them. One study, for example, established that astrocytes secrete ectosomes that are rich in cytokines and other pro-inflammatory molecules, suggesting the action of EVs as neuromodulators. 19 As mentioned earlier, oligodendrocytes also secrete extracellular vesicles. Previous studies have proven that these cells secrete EVs which provide neurons with structural and trophic support. 14 20 A specific study pointed out an interesting result about a possible function of the EVs secreted by oligodendrocytes. 21 In this work it was found that primary rat neurons cultured in a culture medium rich in oligodendrocyte-derived EVs had more active metabolism in ischemic situations (lack of glucose and oxygen) than those not grown in this medium. This result is probably due to the transfer of the enzymes superoxide dismutase and catalase to the neurons via oligodendrocyte-derived EVs (ODEVs). In addition, one study performed a proteomic analysis of ODEVs. 14 In this analysis, the presence of important proteins for myelin formation was found, such as myelin basic protein and myelin oligodendrocyte glycoprotein, suggesting that ODEVs may play a role in the regulation of myelin sheath formation. Extrapolating their physiological functions, several studies point out that EVs may be related to the pathogenesis of various diseases, contributing to them, or acting as a reflection of cellular stress and/or injury, which allows their use as biomarkers. 22 Among the diseases in which it is thought that EVs may play an important role, one of the most researched is cancer. Studies have shown that the metastasis of tumor cells is preceded by the elimination of EV's by tumor cells and that these EV's would have the function of preparing the distant tissue for metastasis to be successful. 23 24

## EXTRACELLULAR VESICLES AS BIOMARKERS IN ALZHEIMER'S DISEASE

The role of EVs in the pathophysiology of neurodegenerative diseases such as Alzheimer's, Parkinson's, and prion diseases has been the subject of recent studies, due to the fact that these diseases share a similar mechanism, in which there is misfolding of certain proteins, their deposition and the subsequent dissemination to specific regions of the CNS. Since EVs play a role in cell signaling, it is thought that they may be a vehicle for the transmission of the misfolded proteins, thus contributing to the pathogenesis of the above diseases. 25

Alzheimer's disease (AD) is the most common neurodegenerative disease affecting the CNS, leading to neuronal death. When the clinical diagnosis is made, the disease is quite advanced and a large neuronal loss is already established, making the treatment possibilities not so effective. It is in this context that EVs may become important: recent studies indicate the possibility of using them as early diagnostics biomarkers of AD, enabling a more effective treatment in individuals diagnosed at earlier stages. 26 The pathophysiological changes in AD are accompanied by changes in both the quantity and composition of neuron-derived EVs (NDEVs), neuron precursor cells, and astrocytes. 27 28

## TAU AND Aß

It has been shown that alterations in the neural synapse process modify the secretion of EVs vesicles by neurons. 29 30 Furthermore, the presence of the most predisposing allele to AD, the APOE-ε4 allele, is related to decreased secretion of brain-derived EVs. 31 Aß(Amyloid beta) and tau are protein forms known to be involved in the pathophysiology of AD. 32 33 Aß is an anomalous insoluble protein formed by the differential cleavage of amyloid precursor protein(APP) by the enzymes β-secretase and γ-secretase in the brain of AD patients. Such cleavage leads to the formation of insoluble peptide fragments (Aß40/Aß42) that undergo oligomerization and subsequent polymerization in the synaptic environment, generating deleterious effects, such as alterations in calcium metabolism and energy metabolism, increased oxidative stress, and ion channel blockade, which contribute to the neuronal death seen in the disease. 34 35 Another toxic effect of extracellular amyloid plaque formation is the increase in the pool of protein kinases, such as Glycogen Synthase kinase 3 (GSK3) and Cyclin-dependent kinase 5 (CDK5). These proteins are responsible for the hyperphosphorylation of tau protein fragments, leading to their aggregation and consequent formation of intracellular neurofibrillary tangles(NFTs). 33 Since tau protein, in association with tubulin, is essential for microtubule homeostasis, 36 it is reasonable to infer that interneuronal communication will be affected in the case of NFT formation, which also favors neuronal loss. 37 There is accumulated evidence pointing towards the use of both of this proteins (Aß and tau) as biomarkers of Alzheimer's Disease. More specifically in cerebrospinal fluid (CSF), it is thought that low levels of Aß42 are found due to increased deposition of Aß plaque in the encephalon, whereas a high level of tau and p-tau indicates a high degree of tau protein disturbances. 38 39 40 41 In a murine model of AD, tau protein was shown to spread through the secretion of exosomes, especially by microglia cells, which was postulated after it was discovered that the depletion of these cells decreased such spread, suggesting that inhibition of EV secretion was beneficial in decreasing the spread of defective tau. 42 Furthermore, studies point to the possibility that EV transport of Aß protein in its oligomeric and neurotoxic form through neurons, which is due to the discovery that EVs can carry such protein from brain tissue analysis. These EVs were shown to be deleterious to the primary culture of neurons, demonstrating, *in vitro* , a possible action of EVs in the pathogenesis of the disease, through the transmission of Aß in its neurotoxic form through the CNS. 43

Several studies investigating the levels of Aß42 in neuron-derived EVs isolated from plasma have reported increased amounts of this protein in individuals with AD and mild cognitive impairment (MCI) compared to healthy controls. 44 45 46 47 One study in particular proved that this increase was present up to 10 years prior to AD diagnosis, as well as that these levels increased as the disease progressed. 47 This finding indicates that analysis of Aß42 levels in NBEVs isolated from plasma can serve as both an early biomarker, as well as a biomarker of AD progression. In this case, the use of plasma as the medium for EV extraction is preferred over CSF because the latter method is more invasive, as well as because a recent study has found that the number of EVs in plasma exceeds that found in cerebrospinal fluid. 48

Regarding the analysis of the presence of tau protein - another important component of Alzheirmer's pathophysiology - within neuron-derived EVs in AD, there is not enough conclusive evidence so far. Some studies pointed to high levels of p-tau in individuals with AD-associated dementia compared with controls, levels which reached a *plateau* about 10 years before clinical diagnosis, 44 46 47 which indicates that p-tau analysis may be less efficient than Aß42 analysis with respect to the investigation of disease progression. On the other hand, three studies found no significant difference in the amount of p-tau in NDEVs in AD. 45 49 50

## NDEVs CONTAINING OTHER PROTEINS INVOLVED IN AD PATHOGENESIS

Tau and Aß are not the only proteins found in neuron-derived EVs that can be used as biomarkers for Alzheimer's. One study demonstrated that the levels of some synaptic proteins such as neurogranin, synaptotagmin, synaptopodin, and synaptophysin, were reduced in the EVs isolated from the plasma of individuals with AD. However, these proteins are also reduced in individuals with MCI and Parkinson's, so that their selectivity is not as high. 51 SNAP-25 and synapsin 1 proteins also have reduced levels in NDEVs isolated from the blood of AD patients compared to control subjects. 51 52 One study compared the levels of pre(neuronal pentraxin 2, neurexin 2 alpha) and postsynaptic proteins (GluA4-containing glutamate receptor, neuroligin 1) in NBEVs of AD subjects and normal subjects. 53 As a result, the levels of the four aforementioned proteins were reduced in NDEVs from AD patients, and the levels of the postsynaptic proteins were directly related to cognitive loss. Also in this study, a longitudinal design was conducted, in which it was found that reductions in the levels of these proteins, except for neuronal pentraxin 2, were already observed 6-11 years before diagnosis, and that this was directly proportional to disease progression, indicating a possible use of these proteins as both early biomarkers and biomarkers of AD progression.

Furthermore, insulin metabolism-related proteins are found in NDEVs. Their use as biomarkers of AD has been recently proposed, based on the finding that increased levels of phospho-Ser312-IRS1 and decreased phospho-panTyr-IRS-1 were found in individuals with AD. 54 55 Lysosomal proteins found in NDEVs have also been considered as possible early biomarkers of AD. In the study of Goetzl, et al. 2015, 56 levels of cathepsin D, a lysosome-associated membrane protein, and ubiquitinated proteins were increased, whereas heat-shock protein-70 levels were decreased in individuals in the preclinical and clinical stages of AD.

## EVs FROM OTHER CELL TYPES

As previously stated, it is possible to purify EVs derived from several CNS cells, not only neurons. One study analyzed the content of EVs derived from a specific type of neuron precursor cell: the CSPG4 cells (Chondroitin sulfate proteoglycan). These cells have the function of secreting substances that are determinants for the growth and survival of neurons. In this sense, EVs were found to have significantly lower levels of four neurotrophic factors (hepatocyte growth factor, fibroblast growth factors 2 and 13, and type 1 insulin-like growth factor) in AD patients in preclinical phase, exposing the possibility of using these proteins as early biomarkers of AD. 57 Astrocyte-derived EVs also have potential biomarkers for AD. One study 58 found elevated levels of BACE1 (a protein that initiates pathological APP cleavage in Alzheimer's pathophysiology), sAPPß and complement proteins, while another study found low levels of glia-derived neurotrophic factor (GNDF) in individuals with AD. 59

## ANALYSIS OF EV CARGO

The analyses of the genetic content in EVs may be an important way of obtaining biomarkers for AD as well as in several other disease conditions. It has been shown that exosomes carry mRNA and microRNAs (miRNAs) in addition to proteins and other macromolecules, 60 and abnormalities of miRNA expression have been found in exosomes from individuals with AD. 61 Some studies have already demonstrated differences in the expression of various types of miRNAs in exosomes purified from the plasma or serum of individuals with AD and MCI. 29 62 63 64 As an example of this, studies have found that the expression of the exosome-associated miRNAs miR-342-3p,miR-125a-5p, miR-125b-5p, and miR-451a, was significantly lower in individuals with AD, and a correlation can be made between the differences in expression and the level of cognitive defect. 29 65 Interestingly, a study 64 pointed out that the combined analysis of the exosomal expression of miRNAs miR-135a, miR-193b, and miR-384, related to the modulation of APP and/or BACE1 expression, is a good biomarker of early AD. In this sense, one may suggest that a combined analysis of several molecules may be more valuable than the isolated analysis of possible biomarkers for a possible early diagnosis.

A recent study analyzed the content of small nuclear-RNAs(snRNAs) in plasma-derived EV's and their use as a biomarker of AD. 66 Levels of 4 snRNAs: miRNAs(microRNAs), snoRNAs (small-nucleolarRNAs), tRNAs (transferRNAs) and piRNAs (piwi-interactingRNAs) were analyzed via droplet-digital-PCR(ddPCR). The results showed that there was significantly different expression of two snoRNAs, SNORD115 and SNORD 116, both of which are predominantly expressed in the brain. 67 68 69 70 71 Such differential expression of these two snoRNAs in EVs isolated from the plasma of individuals, when analyzed combined, enabled the differentiation of AD from healthy controls(Area under the curve of 94.7%), higher than other biomarkers. Another paper 72 aimed to ascertain whether there are differences in mitochondrial RNA (mtRNA) content in EVs isolated from plasma between AD, MCI, and controls (age-matched) individuals. Using the RNA-seq technique, the researchers confirmed significantly elevated levels of certain mitochondrial RNAs (MT-ND1-6, MT-ND4L, MT-ATP6, MTATP8, MT-CYTB, MT-CO1, MT-CO2, MT-CO3 mRNAs, and MT-RNR1 rRNA) in EVs isolated from the plasma of AD and MCI. The researchers proposed that since the pathophysiological mechanisms of AD cause mitochondrial damage, 73 74 there is secretion of mitochondrial components through EVs, opening up the possibility for its use as a diagnostic and prognostic biomarker. It is important to mention that in this study EVs were not isolated from CNS cells, since the researchers pointed out that the isolation methods resulted in a very low pool of EVs, which would impair the quality of the study. However, the researchers performed an experiment in which they cultured several CNS cells (microglia, astrocytes, and neurons), imposed on them conditions similar to those seen in AD (Aß42 and ROS), and found EVs containing high levels of mt-RNAs compared to control cultures.

EVs contain, besides proteins and genetic material, lipids, both in the constitution of their membrane and cargo, so it may be possible to use the dosage of these lipids as a possible biomarker of AD. A specific study 75 aimed to investigate whether there are differences in the expression of lipids in Brain-Derived Extracellular Vesicles(BEDVs) between AD and age-matched control individuals, more specifically coming from the frontal cortex tissue of the participants. The study found increased plasmalogen glycerophosphoethanolamine and decreased polyunsaturated fatty acyl-containing lipids in AD BDEVs. All the above information regarding the molecules cited in this article and their potential as biomarkers is summarized in Table 1 .

**Table 1:** Main molecules and findings

Molecules	Findings
Aß42	- reduced levels in cerebrospinal fluid(CSF) of AD patients. 39 - elevated levels in plasma isolated NDEVs from AD an MCI in comparison to controls. 44 46 47 51 - elevated levels in plasma isolated NDEVs from AD in comparison to controls up to 10 years prior to clinical diagnosis, with progressive increase of such levels along with disease progression. 47
Tau	- inconclusive.
Synaptic proteins	
Neurogranin, Synaptotagmin, Synaptopodin and Synaptophysin	- decreased levels in plasma isolated EVs from AD patients in comparison to controls(same in MCI and Parkinsons Disease). 51
SNAP-25 and Synapsin-1	- decreased levels in plasma isolated EVs from AD patients in comparison to controls. 51 52
Neuronal pentraxin-2, Neurexin-2-α, GluA4 **-** containing Glutamate receptor and Neuroligin-1	- reduced levels in plasma isolated NDEVs from AD patients in comparison to controls; the reduced levels correlated with cognitive loss; - the decreased levels of Neurexin-2-α, GluA4-containing Glutamate receptor and Neuroligin-1 were present up to 6-11 years before clinical diagnosis, and reduction was directly proportional to disease progression. 53
Insulin metabolism proteins	
Phospho-Ser312-IRS1	- elevated levels in NDEVs isolated from AD patients plasma. 54 55
Phospho-panTyr-IRS1	- decreases levels in NDEVs isolated from AD patients plasma. 54 55
Lysossomal proteins	
Cathepsin D	- increased levels in NDEVs isolated from AD and pre-clinical AD patients in comparison to controls. 56
Heat shock protein-70	- reduced levels in NDEVs isolated from AD and pre-clinical AD patients in comparison to controls. 56
Neurotrophic factors	
Hepatocyte growth factor, Fibroblast growth factor type 2, Fibroblast growth factor type 13 and Insulin-like growth factor type 1	- reduced levels in neuron precursor cells(CSPG4) derived EVs isolated from the plasma of pre-clinical AD patients in comparison to controls. 57
Astrocytes	
BACE-1 and sAPPß	- elevated levels in astrocyte-derived EVs isolated from AD patientes in comparison to controls. 58
Glia-derived neurotrophic factor(GNDF)	- reduced levels in astrocyte-derived EVs isolated from AD patients in comparison to controles. 59
Micro-RNAs	
miR-342-3p, miR-125a-5p, miR-125b-5p and miR-451a	- reduced expression levels in exossomes isolated from AD patients in comparison to controls; expression levels directly correlates to cognitive defect. 29 65
miR-135a, miR-193b, miR-384	- combined analyses of expression in exossomes is a good biomarker for early AD. 64
Small-nucleolar-RNAs	
SNORD115 and SNORD116	- increased levels in plasma isolated EVs from AD patients in comparison to controls; enabled diferentiation between controls and AD with a 94.7%AUC. 66
Mitochondrial-RNAs	
MT-ND1-6, MT-ND4L, MT-ATP6, MTATP8, MT-CYTB, MT-CO1, MT-CO2, MT-CO3 mRNAs, and MT-RNR1 rRNA	- elevated levels in plasma isolated EVs from AD and MCI patients in comparison to controls. 72
Lipids	
Plasmalogen glycerophosphoethanolamine	- increased levels in BDEVs isolated from frontal cortex tissue of AD patients in comparison to controls. 75
Polyunsaturated fatty acyl containing lipids	- decreased levels in BDEVs isolated from frontal cortex tissue of AD patients in comparison to controls. 75

## CONCLUSION AND PERSPECTIVES

Extracellular vesicles (EVs) correspond to entities composed of a phospholipid bilayer, formed from endosomes or from the evagination of the plasma membrane of cells and their classification is still a topic under discussion. 1 2 3 4 These vesicles carry within them different contents, such as genetic material, proteins, lipids, and carbohydrates, which come from the cells of origin of the vesicle, thus reflecting the state of such cells. 5 6 7 EVs are already known to have diverse functions, with their participation in cell signaling being one of the best understood. However, their presence in systemic processes has already been verified, as well as in several pathologies, such as neurodegenerative diseases, Alzheimer's being the disease addressed by this article. 25 In this sense, several alterations have been verified in both the number and content of extracellular vesicles extracted from patients with Alzheimer's disease(AD), or in animal models. Because EV's can be isolated from various biofluids such as CSF, blood, urine, among others, their use as a biomarker is quite an exciting prospect, allowing better screening and treatment chances for the patients. The aim of the article, therefore, was to gather the previously published information about the use of EVs as biomarkers in AD. Although the information is promising, more studies are needed, especially longitudinal studies, to obtain a reliable pattern of change in the content and number of EVs, a pattern that can be used on a large scale and is very efficient in the early diagnosis of AD, in order to improve the quality of life of patients.
